# Galanin Receptor 1 Deletion Exacerbates Hippocampal Neuronal Loss after Systemic Kainate Administration in Mice

**DOI:** 10.1371/journal.pone.0015657

**Published:** 2010-12-13

**Authors:** P. Elyse Schauwecker

**Affiliations:** Department of Cell and Neurobiology, University of Southern California Keck School of Medicine, Los Angeles, California, United States of America; Boston University School of Medicine, United States of America

## Abstract

**Background:**

Galanin is a neuropeptide with a wide distribution in the central and peripheral nervous systems and whose physiological effects are mediated through three G protein-coupled receptor subtypes, GalR1, GalR2, and GalR3. Several lines of evidence indicate that galanin, as well as activation of the GalR1 receptor, is a potent and effective modulator of neuronal excitability in the hippocampus.

**Methodology/Principal Findings:**

In order to test more formally the potential influence of GalR1 on seizure-induced excitotoxic cell death, we conducted functional complementation tests in which transgenic mice that exhibit decreased expression of the GalR1 candidate mRNA underwent kainate-induced status epilepticus to determine if the quantitative trait of susceptibility to seizure-induced cell death is determined by the activity of GalR1. In the present study, we report that reduction of GalR1 mRNA via null mutation or injection of the GalR1 antagonist, galantide, prior to kainate-induced status epilepticus induces hippocampal damage in a mouse strain known to be highly resistant to kainate-induced neuronal injury. Wild-type and GalR1 knockout mice were subjected to systemic kainate administration. Seven days later, Nissl and NeuN immune- staining demonstrated that hippocampal cell death was significantly increased in GalR1 knockout strains and in animals injected with the GalR1 antagonist. Compared to GalR1-expressing mice, GalR1-deficient mice had significantly larger hippocampal lesions after status epilepticus.

**Conclusions/Significance:**

Our results suggest that a reduction of GalR1 expression in the C57BL/6J mouse strain renders them susceptible to excitotoxic injury following systemic kainate administration. From these results, GalR1 protein emerges as a new molecular target that may have a potential therapeutic value in modulating seizure-induced cell death.

## Introduction

Epilepsy is a chronic neurological disorder characterized by the occurrence of spontaneous recurrent seizures, which consist of prolonged and synchronized neuronal discharges. The most common form of epilepsy is temporal lobe epilepsy (TLE), a catastrophic disorder characterized by pharamacologically intractable seizures and progressive cognitive impairment. Hippocampal sclerosis, a pattern of neuronal loss in vulnerable mesial structures of the temporal lobe, is found in about 70% of TLE patients [1], [2], and is characterized by severe segmental neuronal loss in the hippocampal subfields CA1, CA3, and the hilar region [3], [4]. TLE is currently considered to be a multifactorial disease, with multiple genetic susceptibility genes implicated and complex gene-environment interactions [5], [6] interplaying to determine disease onset and progression. In addition, the molecular mechanisms involved in the pathogenesis of hippocampal sclerosis remain highly obscure.

TLE-associated brain damage is caused by persistent and highly repetitive seizures that are associated with excitotoxic cell death mechanisms. Excitotoxicity refers to a process of neuronal death initially triggered by elevated levels of excitatory amino acids resulting in the opening of glutamate receptor-associated ion channels causing prolonged depolarization of neurons [7]–[14]. While recent genetic discoveries have led to significant insight into molecular pathways of likely importance in epilepsy pathogenesis [15], these discoveries have not contributed to an understanding of molecular mechanisms that result in seizure-induced cell death. Moreover, host genetic factors may also be important but basic research is lacking with regard to the contributions of genetic variants to seizure-induced cell death.

Previous studies in our laboratory had determined that resistance to excitotoxic cell death varies among mouse strains and some of this variation is assumed to have a genetic basis. We have identified two strains of mice (C57BL/6J and FVB/NJ) that differ in both their genotype and exhibit a maximum difference in susceptibility to excitotoxin-induced cell death [16]–[18]. Although C57BL/6J (B6) and FVB/N (FVB) mouse strains exhibit comparable seizure activity following systemic administration of kainic acid (KA), B6 mice show essentially no hippocampal cell death. These findings suggest that host genetic factors confer protection against hippocampal damage following seizures in resistant strains. Using these mice, we previously identified and confirmed three significant QTL on chromosome 18, 15, and 4 in the mouse genome, responsible for seizure-induced cell death susceptibility through the creation of reciprocal congenic strains and interval-specific congenic lines of mice [19], [20]. The strongest and most significant QTL that determines susceptibility is located on Chr 18 and previous studies have identified galanin receptor type 1 (GalR1) as a compelling candidate gene for the locus on Chr 18 based on expression analyses [21] and its known role as a neuroprotective factor for the hippocampus.

To date, a number of molecular targets have been suggested as anti-excitotoxic agents. Drugs targets that have been shown to modulate glutamate excitotoxicity have included target neurotransmitter receptors, neurotrophins, and more recently, the neuropeptides [22]–[24]. Galanin, a 29 amino-acid secreted neuroactive peptide is widely expressed throughout the peripheral and central nervous systems and is involved in numerous physiological and pathological neuronal functions, such as learning and memory [25], mood [26], [27] and pain control [28], [29], feeding behavior [30], [31] and neuronal protection [32]–[36]. In particular, galanin's neuroprotective effects are thought to occur via modulation of neuronal excitability in the hippocampus and these effects are mediated via three G-protein coupled receptors, namely GalR1, -R2, and –R3 [37]–[39]. Recent evidence indicates that galanin is a potent and effective modulator of neuronal excitability in the hippocampus [40], [41] in that both gain- and loss-of-function experiments have indicated a role for galanin in protection against glutamate toxicity. These studies as well as others [42] confirm that manipulation of galanin expression can modulate hippocampal susceptibility to excitotoxic-induced neuronal damage [43]. Together with these findings, the observation that galanin can modulate neuronal excitability in the hippocampus by inhibiting evoked glutamate release [44] has led to the hypothesis that galanin can act as a neuroprotective factor.

A classical paradigm of excitotoxic cell damage can be produced by systemic administration of the glutamate analogue, kainic acid (KA) [45]–[50]. In order to test more formally the potential influence of GalR1 on seizure-induced excitotoxic cell death, we have conducted functional complementation tests in which we determined if there are functional differences in susceptibility to seizure-induced cell death by utilizing transgenic mice that exhibit decreased expression of the GalR1 candidate mRNA. As an alternative approach to the use of knockout (KO) mice, we also utilized intra-hippocampal pre-treatment administration of a GalR1 antagonist to determine if pharmacologic inhibition of GalR1 could modulate susceptibility to seizure-induced cell death. In the present study, we therefore aimed to investigate the role of GalR1 in KA-induced neurodegeneration in C57BL/6J mice. Our results demonstrate that deficiency of GalR1 exacerbates seizure-induced neuronal death after KA-induced excitotoxicity in the brain. These results imply a critical role for both GalR1 as well as the galanin cascade itself as mediators of post-seizure-induced damage. While the underlying mechanism is unclear, its elucidation is likely to promote our understanding of the neurodegenerative process in temporal lobe epilepsy.

## Results

### Characterization of GalR1 KO mice

**Figure 1:**
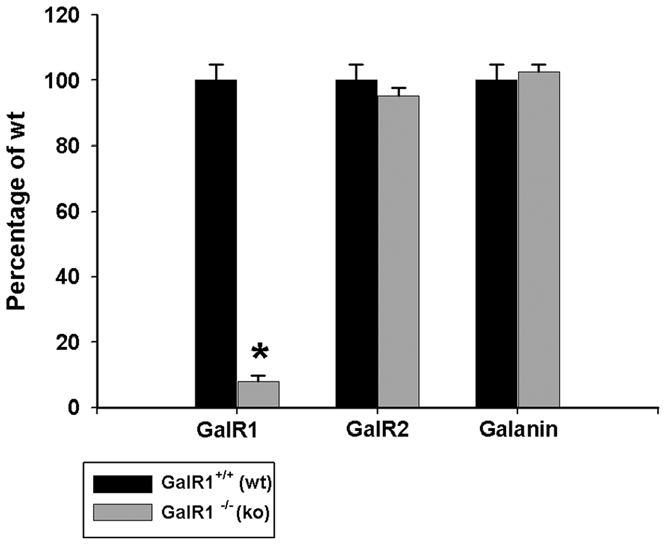
Characterization of mice lacking GalR1. Real-time PCR quantification of the levels of GalR1, GalR2, and galanin produced in the hippocampus of wildtype (GalR1^+/+^) and GalR1 mutants (GalR1^−/−^). Expression levels were standardized relative to GAPDH transcript levels using the standard curve method. Values are provided as mean ± SEM from 6–9 mice per strain analyzed in triplicate. Expression studies demonstrated that the hippocampus of knockout animals had a minimal expression of GalR1 when compared to wild-type littermates. In contrast, differences in the expression of GalR2 or galanin between GalR1^−/−^ mice and their wildtype littermates were not observed in the hippocampus. *P<0.05.

To ensure that compensatory alterations in GalR2 or galanin did not exist in GalR1 null mutant mice (GalR1^−/−^), we used QRT-PCR to measure levels of GalR2 and galanin in the hippocampus of GalR1^−/−^ mice and their respective wildtype littermates (GalR1^+/+^). We also wanted to confirm the absence of GalR1 in GalR1^−/−^ mice. Analysis of mRNA from hippocampus of GalR1^−/−^ mice confirmed the absence of normal full-length transcript encoding GalR1 (Fig. 1). In contrast, we found no evidence for differences between GalR1^−/−^ mice and their wildtype (WT) controls (GalR1^+/+^) in the expression of GalR2 or galanin in the hippocampus. We thus show normal galanin and GalR2 receptor levels in GalR1 null mutant mice, suggesting that functional loss of one receptor (e.g. GalR1) does not trigger compensatory changes in other receptor levels. As well, any modifications in seizure-induced cell death we observe in GalR1 null mutant mice will likely not be due to adaptive changes in GalR2 or galanin.

### Effect of GalR1 genotype on seizure parameters

**Figure 2:**
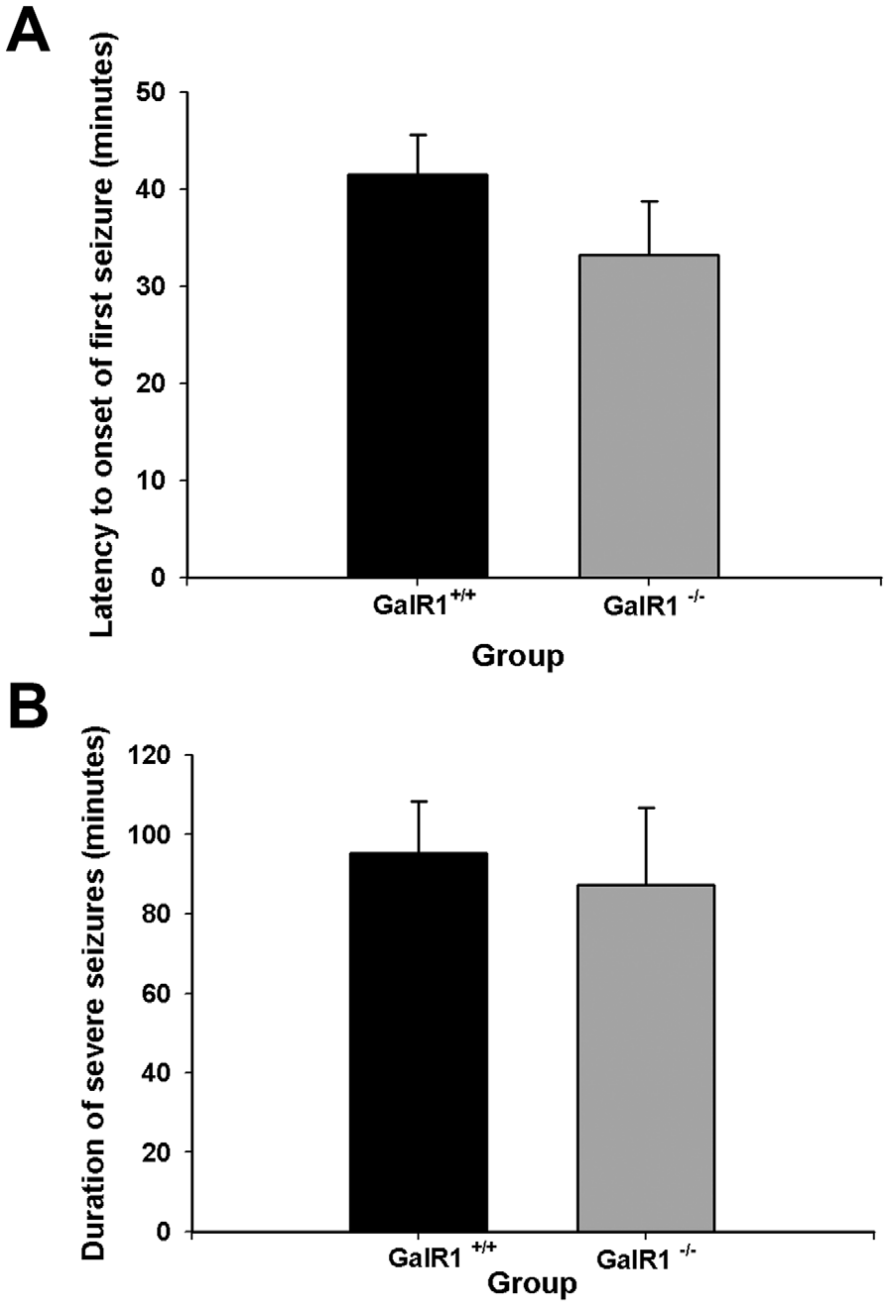
Histograms of seizure parameters in GalR1 mutant mice following kainic acid-induced status epilepticus. (A) Data represent latency to severe stage 4/5 seizures in minutes (mean ± SEM) for GalR1 ^+/+^ and GalR1 ^−/−^ mice. No significant differences between groups were observed. (B) Duration of severe stage 4/5 seizures in minutes for GalR1 ^+/+^ and GalR1 ^−/−^ mice was not significantly different among mice. Data represent the mean ± SEM of at least 6 mice per group. *P<0.05.

Kainic acid (KA) is a potent neurotoxin that, when injected systemically, produces epileptic behavior and subsequent neurodegeneration [50]. A systemic injection of KA into both GalR1^+/+^ and GalR1^−/−^ mice produced the development of typical seizure behavior. Approximately, 15 min after the KA injection, all mice displayed a series of behavioral stages of seizures as previously reported [17], [18], [51]. In brief, mice initially assumed a catatonic phase and were immobile. This was followed by forelimb clonus and hindlimb clonus within 20–30 min after injection. By 35–45 min after injection, all mice were rearing and exhibited continuous tonic-clonic seizures that lasted ∼1.5 h. No qualitative differences in seizure intensity (data not shown) or latency to onset of severe seizures (Stage 4/5-Racine) were observed between GalR1^−/−^ and GalR1^+/+^ mice after KA administration (Fig. 2A). As well, we found no significant difference in the duration of severe seizures (Stage 4/5-Racine) after KA (Fig. 2B). Saline-treated mice of both genotypes showed no signs of epileptic activity.

### GalR1 deficiency exacerbates KA-induced neuronal degeneration

**Figure 3:**
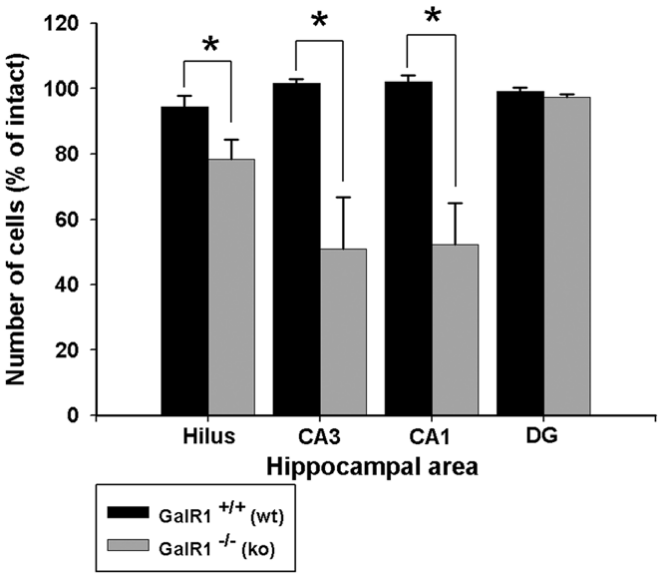
Kainate-induced cell death is substantially increased in GalR1^−/−^ mice. Quantitative analysis of neuronal density in four hippocampal subfields following kainate-induced status epilepticus to young adult mice. Viable surviving neurons were estimated by cresyl violet staining. Bars denote the percentage of surviving neurons (as compared with saline-injected control mice) in each hippocampal region. Differences in the extent of cell loss in three hippocampal subfields were observed 7 days following kainate administration between the GalR1^−/−^ and GalR1^+/+^ mice. Data represent the mean ± SEM of at least 6 mice/condition. CA1 and CA3, hippocampal subfields; Hilus, dentate hilus; DG, dentate gyrus. *P<0.05.

**Figure 4:**
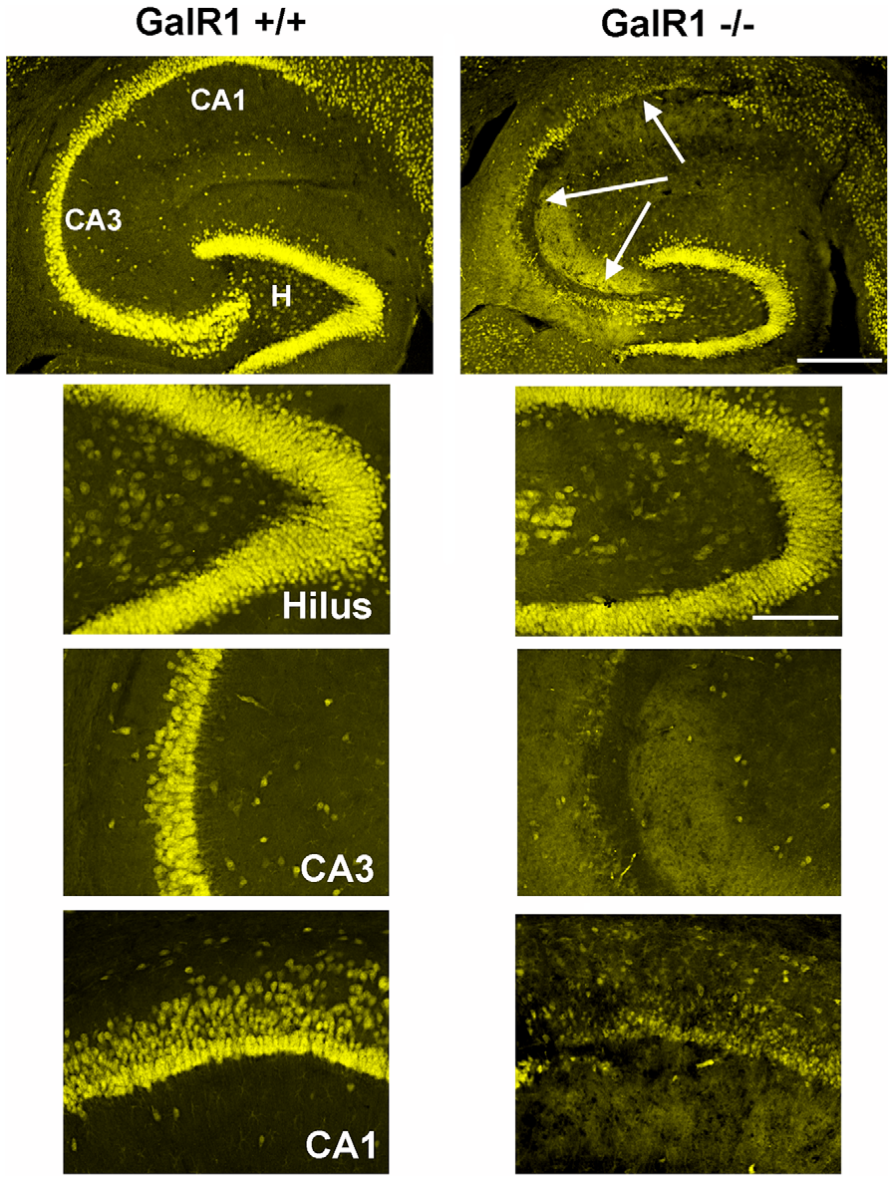
GalR1 deficient mice show increased susceptibility to seizure-induced cell death. Corresponding low-power and high-power photomicrographs of NeuN-immunofluorescent stained horizontal sections of the hippocampus depicting surviving cells throughout the hippocampus 7 days following systemic KA administration to GalR1^−/−^ and GalR1^+/+^ mice. Hippocampal sections from GalR1^+/+^ and GalR1^−/−^ brains were stained with NeuN immunofluorescence to determine the amount of cellular damage. Note the massive loss of neurons, as evidenced by loss of immunostaining, in the hilar, CA3 and CA1 fields of the hippocampus, seven days after a systemic injection of KA inGalR1^−/−^ mice. In contrast, hippocampal cell death was essentially non-existent throughout all hippocampal subfields in GalR1^+/+^ mice. CA1 and CA3 denote the hippocampal subfields; H, dentate hilus. Scale bars = 750 µm (top panels); 100 µm (bottom panels).

Consistent with previous studies in C57BL/6J mice [16]–[18], KA administration into GalR1^+/+^ mice generated very little degeneration. In contrast, KA administration into GalR1^−/−^ mice generated pronounced neurodegeneration and cell loss in the CA3 and CA1 areas of the hippocampal formation, and within the dentate hilus (Figs. 3,4), 7 days following kainate administration. The 7-day survival time was chosen to allow sufficient time for alterations in the rate of maturation of cell death, which could be influenced by gene deletion. In agreement with previous studies [49], [52]–[54], cells within the dentate granule cell layer and area CA2 of Ammon's horn were spared. Sections from these two groups of mice (GalR1^−/−^ and GalR1^+/+^), sacrificed 7 days following kainate injection and processed for NeuN immunofluorescence are shown in Figure 4. As demonstrated in Fig. 3, following kainate administration to GalR1^−/−^ mice, a dramatic reduction of pyramidal neurons in area CA3 and area CA1 was observed. Similarly, a significant reduction was also noted within the dentate hilus. In contrast, representative sections from the GalR1^+/+^ mice showed a significant attenuation in the extent of cell loss throughout all hippocampal subfields. These data indicate that genetic deletion of the GalR1 receptor increased the neuronal loss in the CA3 and CA1 regions, as well as the dentate hilar region, of the hippocampus after systemic kainate administration.

Quantitative analysis of subfield group means revealed that GalR1^−/−^ mice displayed a reduction of 22% of dentate hilar neuron profiles, 42% of CA3 pyramidal neuron profiles, and 45% of CA1 pyramidal neuron profiles after KA administration (F = 5.928; P<0.001; Fig. 3) as evidenced by decreased cresyl violet staining and decreased NeuN-immunostaining (Fig. 4). In contrast, GalR1^+/+^ mice displayed no detectable evidence of degenerative debris or reduction in neuronal profiles in any of the hippocampal subfields after KA administration (Fig. 4).

### Effect of GalR1 antagonist administration on seizure parameters

**Figure 5:**
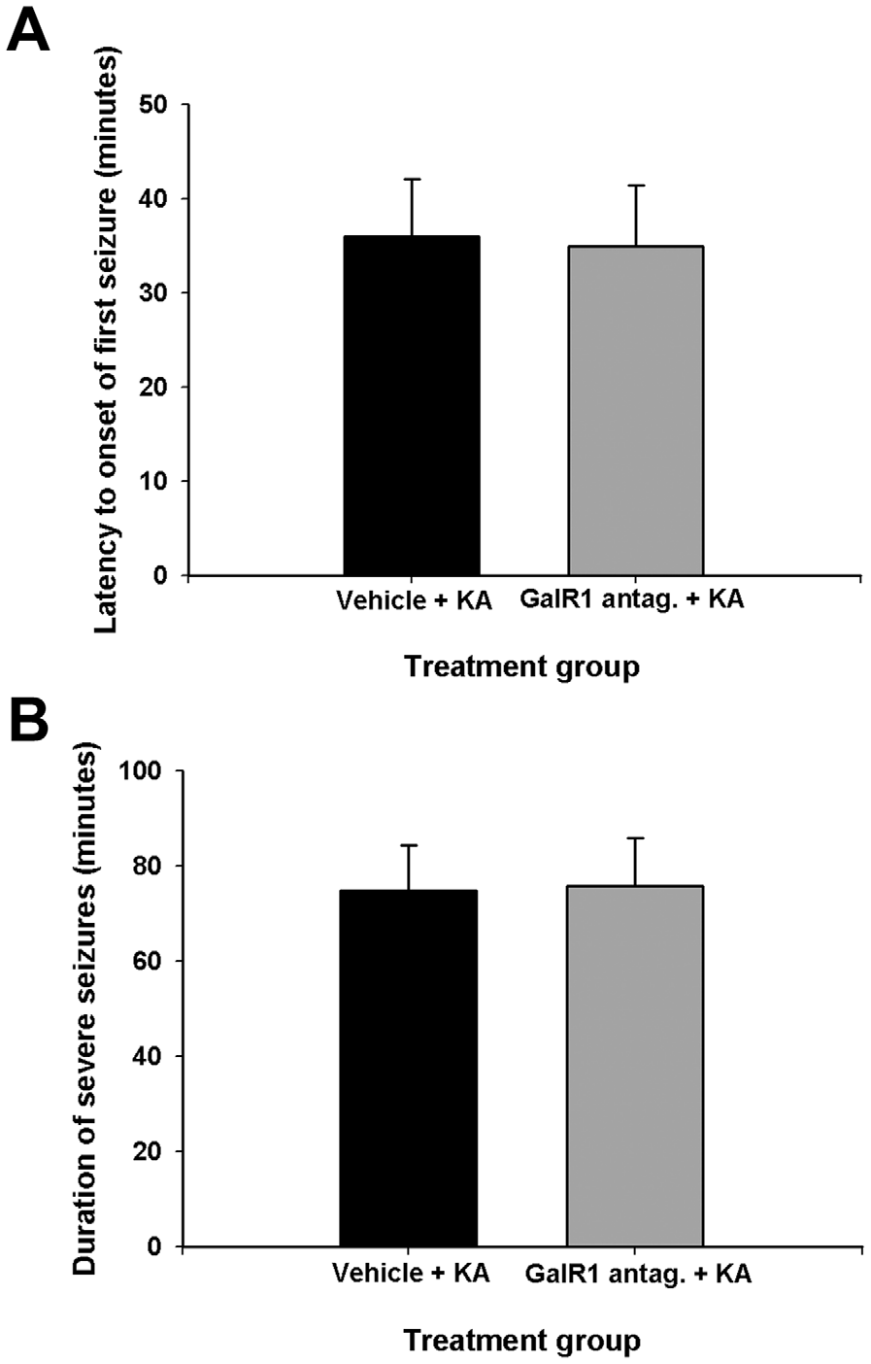
Histograms of seizure parameters in vehicle-treated versus GalR1 antagonist-treated mice following kainic acid-induced status epilepticus. (A) Data represent latency to severe stage 4 seizures in minutes (mean ± SEM). No significant differences between treatment groups were observed. (B) Duration of severe stage 4 seizures in minutes was not significantly different among GalR1 antagonist- versus vehicle-treated mice. Data represent the mean ± SEM for at least 8 mice for each condition. *P<0.05.

We have utilized an antagonist to GalR1 as a pre-treatment prior to kainate administration as a potential alternative approach to the utilization of GalR1 null mutant mice. Administration of KA induced seizures in both galantide- and vehicle-treated mice, with a behavioral progression similar to what we observed in GalR1 null mutant mice. In short, between 35 and 45 min after injection, all mice were rearing and exhibited continuous tonic-clonic seizures that lasted, on average 75±9.6 min. Similar to our observations in GalR1 null mutant mice, neither latency to onset of severe seizures (Stage 4/5-Racine; Fig. 5A) nor duration of severe seizures (Stage 4/5-Racine; Fig. 5B) was modulated by GalR1 antagonist administration. In particular, no qualitative differences in seizure intensity were observed in B6 mice after KA administration with or without galantide administration.

### Pharmacologic inhibition of GalR1 exacerbates seizure-induced cell death

**Figure 6:**
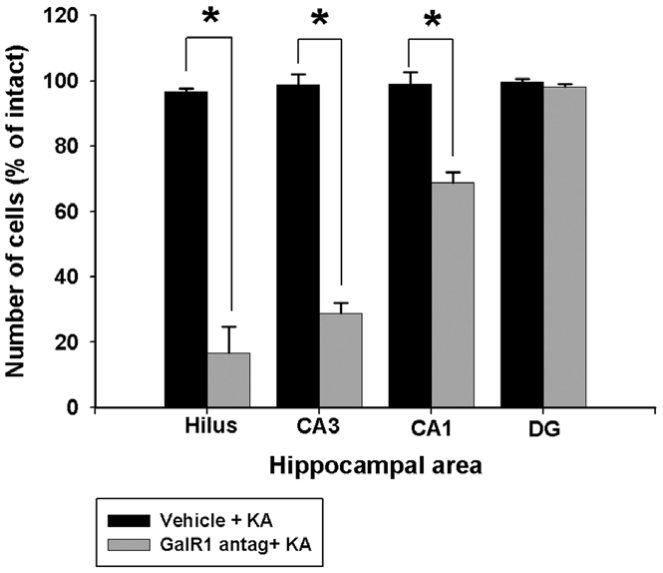
Quantification of kainate (KA)-induced damage in hippocampal subfields in vehicle-injected C57BL/6 (resistant) and GalR1 antagonist treated C57BL/6 mice. Quantitative analysis of neuron density in the dentate hilus, areas CA3 and CA1, and dentate gyrus of C57BL/6 following intrahippocampal administration of 1 µM galantide. Viable surviving neuronal profiles were estimated by cresyl violet staining and counts were performed in each subfield for the most ventral hippocampal sections and averaged into single values for each animal. Bars denote the percentage of surviving neuronal profiles (as compared with saline-injected control mice) in each hippocampal region. Comparison of neuronal profile counts between treatments revealed statistically significant differences when comparing vehicle + KA versus GalR1 antagonist + KA. Data represent the mean ± SEM of eight to 10 mice/treatment. *P<0.05 as compared with intact mice of the C57BL/6 strain.

To determine whether GalR1 activity is required for neuronal death in the model, we examined neurodegeneration in vehicle- and galantide-treated mice. We examined differences in status-induced neuronal injury in young adult B6 mice treated with kainate alone with those that received a single intra-hippocampal injection of the GalR1 antagonist, galantide, 45 minutes prior to kainate-induced SE. We found significant differences in the extent of injury between those mice that received the antagonist and those that received vehicle when cell death was measured 7 days later (Fig. 6). In particular, administration of galantide before KA administration resulting in a significant reduction in neuronal profile counts within area CA3 (F = 104.224; P<0.001), the dentate hilus (F = 97.739; P<0.001), and area CA1 (F = 38.932; P<0.001) ipsilateral to the injection of galantide in C57BL/6 mice (Fig. 6). Mice injected with galantide prior to kainate displayed a reduction of nearly 90% of dentate hilar neurons, 72% of CA3 pyramidal neurons, and nearly 30% of CA1 pyramidal neurons at 7 days following KA-induced SE than vehicle-injected controls. SE in galantide-treated mice also significantly increased the severity of neuronal damage ipsilateral to the injection site in several regions, including the amygdala, pyriform cortex, and thalamus (data not shown).

**Figure 7:**
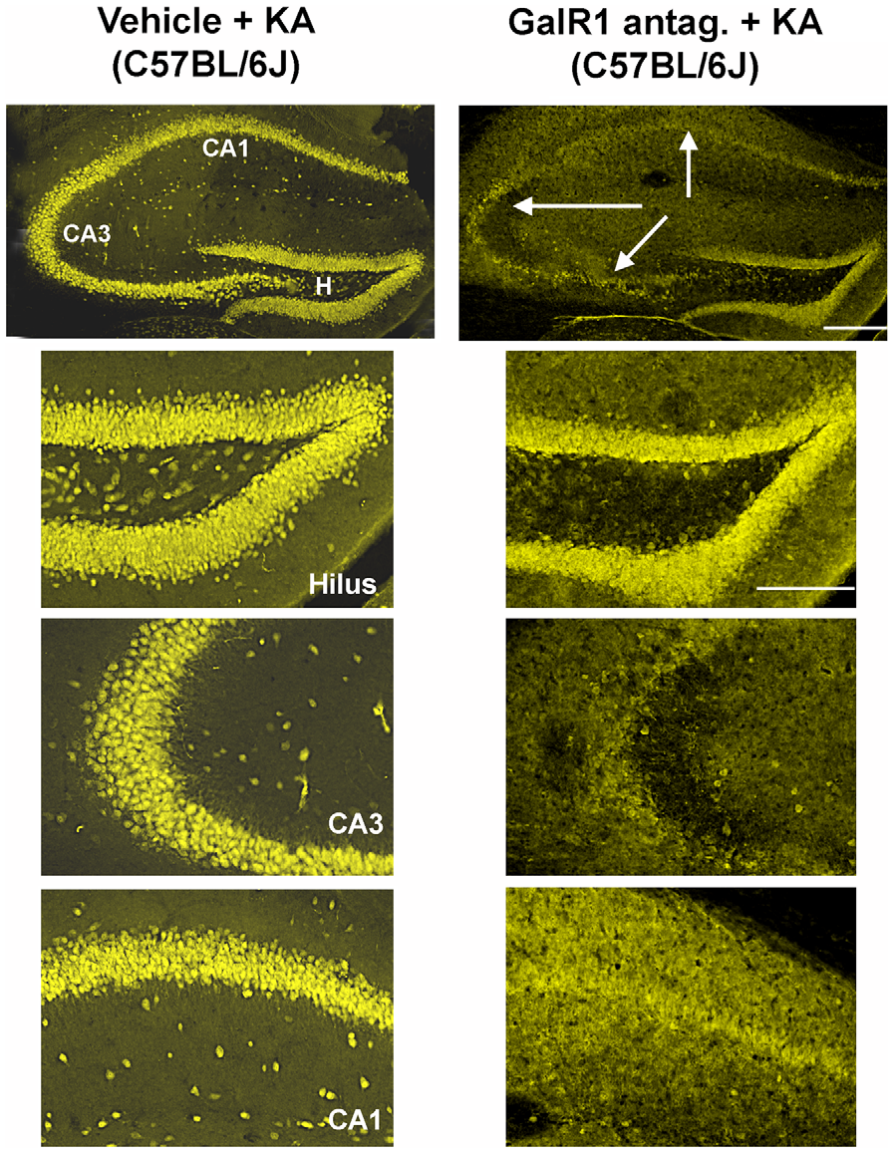
C57BL/6 mice pre-treated with the GalR1 antagonist, galantide, show increased susceptibility to the neurotoxic effects of kainate. Corresponding low-power and high-power photomicrographs of NeuN- stained horizontal sections of the hippocampus showing differential cell loss 7 days after kainate-induced SE (vehicle), and in a representative mouse pre-treated with the GalR1 antagonist, galantide. Note that while hippocampal cell death is essentially non-existent following kainate-induced status epilepticus (Vehicle) to excitotoxin cell death-resistant mice (C57BL/6), a massive loss of neurons, as evidenced by loss of immunostaining in the hilar, CA3, and CA1 fields of the hippocampus, was induced by pre-treatment with galantide prior to KA-induced SE. CA1 and CA3 denote the hippocampal subfields; H, dentate hilus. Scale bars: top panels, 750 µm; bottom panels, 100 µm.

Analysis of hippocampal sections stained with antibodies against NeuN demonstrated that vehicle-treated mice given KA had significantly more surviving neurons after SE, compared to galantide-treated seizure mice (Fig. 7). In addition, no cell loss was noted in the contralateral hippocampus of any galantide-injected animal. In particular, we saw a significant increase in neuronal damage within the dentate hilus, area CA3 and area CA1, of galantide-treated seven days following kainate administration. Vehicle-treated C57BL/6J mice that underwent kainate-induced SE did not show any signs of neuronal damage either within the hippocampus (Fig. 7) or in any extra-hippocampal areas (data not shown).

## Discussion

In the present study, we investigated the role of GalR1 in the pathogenesis of KA-induced excitotoxic neurodegeneration by using GalR1-deficient mice and WT mice to determine whether an imbalance in the galanin receptor system altered neuronal degeneration. The study was motivated by the fact that galanin has been shown to be a potent and effective modulator of neuronal excitability in the hippocampus [40], [41], and thus may act as an effective neuroprotective factor. Systemic KA administration has been widely used to study the susceptibility to acute seizures and seizure-induced histopathology in inbred and mutant mouse strains. In mice, the occurrence of brain damage following KA-evoked seizures strongly depends on the genetic background. In particular, certain mouse strains, such as C57BL/6, are resistant to KA-induced cell death [17], [18], [55]. In the present study, wild-type and GalR1^−/−^ mice of a C57BL/6J genetic background were used. Wild-type mice displayed a mild response to seizure-induced cell death, however conversely, GalR1^−/−^ mice displayed high susceptibility to seizure-induced cell death induced by the same dose of KA. This variability in the degree of brain damage following systemic KA administration is in agreement with previous studies [17], [55] and confirms previous observations that GalR1 gene disruption renders the brain more susceptible to status epilepticus-induced brain damage.

Our results show that GalR1 deficiency influences susceptibility to KA-induced injury in C57BL/6J mice, a strain previously shown to be excitotoxin cell death-resistant [17], [18], [55]. Existing data strongly support the concept that GalR1 acts as a neuroprotective factor. Both gain- and loss-of-function experiments have indicated a role for galanin in protection against glutamate toxicity. Enhanced susceptibility to excitotoxin-induced neuronal injury in GalR1 knock-out mice has been observed [42] and galanin overexpression is known to decrease hippocampal neuronal injury resulting from limbic seizures [43], [56], presumably via GalR1 receptor modulation [40]. In particular, Elliott-Hunt et al. [35] demonstrated that an absence of galanin increases the susceptibility to hippocampal cell death using both organotypic and primary dispersed hippocampal cultures although the mechanisms of such protection have not been fully elucidated.

More recently, studies have indicated that GalR2 may also play an important role in the neuroprotective effects of galanin in hippocampal neurons [42], [43], [57], [58]. Thus, we were especially interested in determining if the increase in seizure-induced excitotoxic cell death in our GalR1^−/−^ mice could be the result of compensatory changes in either GalR2 or galanin. However, we found that while analysis of mRNA from the hippocampus of GalR1^−/−^ mice confirmed the absence of normal full-length transcript encoding GalR1, we found no evidence for differences between GalR1^−/−^ mice and their wildtype controls in the expression of GalR2 or galanin in the hippocampus. Thus, we can conclude that any modifications in seizure-induced cell death we observed in GalR1 null mutant mice are likely not due to adaptive changes in GalR2 or galanin.

Our finding that the cell death inducing effects by GalR1 reduction did not affect seizure susceptibility or duration of severe seizures is interesting and is in agreement with previous studies. Mazarati and Lu [40] found that while GalR1^−/−^ mice showed more severe and longer lasting seizures following pilocarpine-induced status epilepticus or perforant path stimulation (PPS), no difference in seizure severity was noted following kainate administration. In particular, GalR1 receptor knockdown seems to increase the extent of seizure-induced cell death, while having no effect on the severity of seizures or duration of seizures. These results suggest that seizure elicitation and cell death susceptibility are regulated in part by different mechanisms. The exact mechanism by which GalR1 receptors modify cell death susceptibility remains unknown, but earlier studies have suggested that the ability of galanin to reduce glutamate release under high levels of neuronal activation [44], [56], [59] may play an important role. Moreover, the absence of behavioral differences between wild-type and mutant mice following KA indicates that the receptor and/or signal transduction mechanisms that trigger seizures are functional in the mutant mice and further, that the increased cell death observed in GalR1^−/−^ mice is not the result of enhanced seizure activity. Thus, GalR1 deficiency does not appear to alter the susceptibility of mice to kainate-induced seizures and does not appear to act as an effective anti-convulsant in the kainate model of limbic status epilepticus.

To confirm the neuroprotective role of GalR1 and as an alternative approach to the utilization of GalR1 null mutant mice, we injected the GalR1 antagonist, galantide, prior to systemic administration of KA. Pretreatment with the GalR1 antagonist, galantide, before systemic KA administration induced a phenotypic switch in inbred strains of mice previously characterized as kainate-induced cell death resistant (C57BL/6J) [17]. These results lend additional support to our findings that the effect of GalR1 receptor deficiency on cell death in the knockout mice was specific. Furthermore, these results suggest that galanergic innervation attenuates KA-induced neuronal damage in the hippocampus through GalR1 receptors. The spatial differences in mRNA expression of galanin receptor subtypes in the mouse hippocampus are worth noting in light of our results. Although GalR1 is the least abundantly expressed subtype in the hippocampus, it is most prominent in the highly vulnerable CA3 neurons. At present, we cannot explain why GalR1 antagonist administration results in increased vulnerability to kainate-induced cell death in C57BL/6 mice. Thus, additional studies must clarify the specific cellular mechanisms of galantide that promote neurotoxicity in excitotoxin cell death-resistant mice. Surprisingly, the injection of galantide induced a more significant extent of cell death than genetic ablation of GalR1. A possible explanation is that the injection of galantide inhibits the actions of GalR1 and downstream intracellular signaling pathways via GalR1.

This study demonstrates that deficiency of GalR1 exacerbates KA-induced excitotoxicity and identifies GalR1 as a target contributing to seizure-induced cell death *in vivo*. Our findings that GalR1 gene-deleted mice are strikingly more susceptible to seizure-induced cell death are in agreement with previous studies [35], [42], [56], and implies a critical role for both GalR1 as well as the galanin cascade itself as mediators of post-seizure-induced damage. While the underlying mechanism is unclear, its elucidation is likely to promote our understanding of the neurodegenerative process in temporal lobe epilepsy. Thus, taken together our findings suggest that GalR1 deletion may contribute to hippocampal injury after kainate administration. In order to truly establish causality, pharmacologic rescue or genetic reconstitution experiments will need to be carried out. As well, studies using conditional GalR1 knockout and conditional GalR1 transgenic animals to assess the effects of decreased and increased Gal activity post-status would also be useful. Alternatively, the development of new and preferably subtype specific galanin inhibitors and activators would be advantageous. The identification of molecules that play a key role in protecting the brain against seizure-associated excitotoxic injury may illuminate new pathways involved in epilepsy as well as hypoxia, stroke and other related pathologies, and may also aid in the discovery of the common human genetic variation that not only plays a role in determining one's genetic susceptibility to epilepsy, but also to CNS recovery following stroke, and other excitotoxic stressors.

## Materials and Methods

### Ethics statement

Animal care and use were in accordance with National Institutes of Health guidelines. All procedures were approved by the University of Southern California Institutional Animal Care and Use Committee under protocol #10313.

### Mice

GalR1 null mutant mice (B6.129P2-Galr1^tm1Dgen/J^) were originally generated and created by Deltagen, Inc. GalR1^−/−^ mice were generated by insertional mutagenesis of the gene encoding GalR1 resulting in the absence of normal full-length transcript on a mixed 129/B6 genetic background [60]. Galanin binding is dramatically reduced in these GalR1^−/−^ mice [61], confirming functional loss of GalR1. The line was then re-derived at the Jackson Laboratory (Bar Harbor, ME), backcrossed onto C57BL/6J for at least six additional generations, and is now maintained on a B6 genetic background. All mice were aged between 6 and 8 weeks when tested and housed under standard conditions on a 12-h light/dark cycle and given ad libitum access to food and water. *All of the procedures used in these experiments were in accordance with the NIH Guide and approved by the USC Animal Care and Use Committee.* All efforts were made to minimize the number and suffering of any animals used in these experiments.

### Genotyping

Genotyping of GalR1 null mutant mice was performed by PCR analysis. Genomic DNA was isolated from mouse tails according to a previously established protocol [62]. Briefly, a small piece (∼1 cm) of the tip of the tail was cut off using sharp scissors. Tail tips were incubated overnight at 55°C in 635 µL of lysis solution containing 600 µL Tris-NaCl-EDTA-SDS buffer (10 mm Tris, pH 7.5, 400 mm NaCl, 100 mm EDTA and 0.6% sodium dodecyl sulfate) and 35 µL proteinase K (10 mg/mL). DNA was isolated using a high-salt method, and standard procedures of ethanol precipitation and resuspension in Tris-EDTA (10 mm Tris, 1 mm Na_2_-EDTA, pH 7.4) and storage at 4°C. Genotypes were determined using allele-specific polymerase chain reaction (PCR). All samples were tested for the presence of wildtype (WT) bands (429 bp) using the GalR1 assay (35 cycles: 94°C for 30 s, 63°C for 30 s and 72°C for 30 s) using a gene-specific primer, GS(E,T), that lies outside of and adjacent to the targeting vector arm (GTTGCTGTCCCGATGGAAAAGACGC), paired in succession with one of the two primers in the insertion fragment, Neo-T (GGGGATCGATCCGTCCTGTAAGTCT) or GS(E1) (TTTGGCCTGATTTTCGCGATCGGCG). The presence of both the Neo and GalR1 bands (625 bp) indicates heterozygosity, whereas the presence of only the GalR1 band indicates wildtype. Amplified DNA fragments were visualized by ethidium bromide staining following 2% agarose gel electrophoresis.

### Reverse transcription-polymerase chain reaction confirmation of GalR1^−/−^

Reverse transcription-PCR was used to confirm the inactivation of the GalR1 gene. Briefly, total RNA was prepared from the hippocampal formation of GalR1^−/−^ and WT mice with Trizol reagent (Invitrogen, Carlsbad, CA) following the manufacturer's instructions. Template cDNA was synthesized using an oligodT primer, random primers and qSCRIPT reverse transcriptase (Quanta Biosciences, Gaithersberg, MD). cDNA was amplified for 40 cycles of amplification of GalR1, an initial denaturation step of 95°C for 10 min, followed by 40 cycles of denaturation at 95°C for 5 s and annealing/extension at 60°C for 30 s and a final extension at 72°C for 45 s with GalR1-specific primers (5′AGCACCACCAACGCTGTTTATC3′ and 5′-CCAGGTGGGCAGTGCATA'3′). Primers were designed with Primer 3 software [63] based on the murine GalR1 and GAPDH sequences obtained from Ensembl genome browser (release 37). All primers were purchased from Sigma Genosys (The Woodlands, TX) as high performance liquid chromatography purified oligos and tested to determine whether they produce a single band on an agarose gel. The sequences of the primers were : GalR1 (5′- AGCACCACCAACGCTGTTTATC-3′ and 5′- CCAGGTGGGCAGTGCATA-3′), which generates a 102 bp amplicon; GalR2 (5′-CCAGCCTGTTAAAGGACCAA-3′ and 5′-ATGGAAGGGCTATGTCXACCA-3′), which generates a 150 bp amplicon, Galanin (5′-GTGACCCTGTCAAGCCACTCT-3′ and 5′-GGTCTCCTTTCCTCCACCTC-3′), which generates a 150 bp amplicon; GAPDH (5′-AACGACCCCTTCATTGAC-3′ and 5′-TCCACGACATACTCAGCAC-3′), which generates a 162 bp amplicon. ThePerfecta SYBR Green PCR Core reagents kit (Quanta Biosciences,Gaithersberg, MD) was used for PCR, which was performed in MicroAmp Optic 96-well reaction plates (Applied Biosystems) on an ABI 7300 real-time PCR system (Applied Biosystems), with the condition of 10 minutes at 95°C, then 40 cycles of 5 seconds at 95°C, 30 seconds at 60°C, and 45 seconds at 72°C. Each study consisted of 3 independent runs of PCR in triplicate, and each run included a standard curve, nontemplate control, and negative RT control. The concentrations of primers were 100 nM (GalR1 and GAPDH) and 250 nM (GalR2 and Galanin). The levels of target gene expression were quantified relative to the level of GAPDH, using the standard curve method. The specificities of the RT-PCR products were confirmed by both a single dissociation curve of the product and a single band with corresponding molecular weight revealed by agarose gel electrophoresis.

### Antagonist administration

Adult male C57BL/6J mice (Jackson Laboratories, Bar Harbor, ME) were anesthetized with tribromoethanol (240 mg/kg, i.p.) and placed in a stereotaxic frame with mouse adaptor and ear bars (David Kopf Instruments, Tujunga, CA). The skin was retracted to expose the skull and a 1-mm-diameter hole was drilled (stereotaxic coordinates: A -2.0 mm from bregma, 1.8 mm lateral from midline, V 2.0 mm from dura) [64]. A 2-µl Hamilton syringe with a 26-gauge needle mounted to an electrode holder containig the GalR1 antagonist, galantide (1 µM; Sigma Aldrich, St. Louis, MO) was inserted directly into the right dorsal hippocampus at the level of the hippocampal CA3 region. An additional group of mice (n = 6) was injected with an equivalent volume (0.5 µl) of saline and served as control subjects. Injections were made over a 5-min time period and the needle was left *in situ* for an additional 5 min and withdrawn slowly to allow the antagonist to diffuse from the needle tip and prevent reflux from the injection site. After recovery from anesthesia and 45 minutes after injection, animals underwent systemic kainate administration.

### Systemic kainate administration

Young adult male mice (GalR1^−/−^, GalR1^+/+^, and C57BL/6J) between 8–10 weeks of age were used in these studies. Sustained seizures (status epilepticus) were induced in animals by the administration of kainic acid (KA), a potent agonist of the AMPA/KA class of glutamate receptors. KA was dissolved in isotonic saline (pH 7.3) and administered subcutaneously to adult mice at a dose of 20 mg/kg. After KA administration, the behavior of each mouse was observed and documented for 3–4 hours to determine the duration and severity of seizure activity using using a previously established six-point seizure scoring scale [17] that was adapted from a five-point scale for rats [65]. The observer was blinded as to whether the animal was a vehicle injected, antagonist injected, wild-type or GalR1 knockout animal. Seizure stages were defined as follows: Stage 1, immobility; Stage 2, forelimb and/or tail extension, rigid posture; Stage 3, repetitive movements, head bobbing; Stage 4, rearing and falling; Stage 5, continuous rearing and falling; and Stage 6, severe tonic-clonic seizures. Only those mice exhibiting at least 45 min of continuous stage 4/5 seizures were included in this study, as previous studies have suggested that there is a direct relationship between the generation of epileptiform activity and the extent of damage in hippocampal subfields [49], [66], [67]. Seizure parameters monitored included latency of convulsions and duration of severe (Stage 4/5) seizure activity. All experiments were approved by the Institutional Animal Care and Use Committee (IACUC) of the University of Southern California and conducted in accordance with its guidelines. Every effort was made to minimize animal suffering and to minimize the number of animals utilized in order to produce reliable scientific data.

### Histologic staining

After a survival time of 7 days, mice received an overdose of tribromoethanol (240 mg/kg, i.p.) and were perfused transcardially with 4% paraformaldehyde in 0.1 M phosphate buffer (pH 7.4) after vehicle + KA, KA, or galantide + KA administration. Brains were immediately removed and postfixed overnight in the same fixative before being cryoprotected in 30% sucrose in 0.1 M phosphate buffer (pH 7.3) for >12–18 h. Brains were frozen onto chucks with embedding medium (Tissue-Tek OCT; Fisher Scientific, Tustin, CA) and surrounded with powdered ice. Horizontal sections (40 µm) through the hippocampus were cut from a dry ice-cooled block on a sliding microtome (Leica, Deerfield, IL) and stored in 0.1 M phosphate buffer (pH 7.4) prior to staining. For histologic assessment, every sixth section was stained with cresyl violet to determine neuronal cell loss and the general histologic features of the tissue. Alternate sections were stained with a modification of the Gallyas silver stain, which stains degenerating fibers, synaptic terminals and cell bodies [68], and examined for the appearance of degenerative debris.

### NeuN immunofluorescence

Immunofluorescence was performed on an additional series of sections (every sixth section; ∼240 µm) to detect those neurons that survived 7 days following kainate-induced SE. For immunofluorescent labeling, sections were washed with 0.1 M phosphate buffer (pH 7.4) and blocked with 5% normal serum and 0.1% Triton X-100 in 0.1 M phosphate buffer (pH 7.4). Next, sections were incubated overnight with a neuronal marker against NeuN (monoclonal from mouse; Chemicon; 1:500) at 4°C. After several washes, sections were incubated with a secondary antibody from mouse conjugated with Cy2 (1:200; Jackson ImmunoResearch, West Grove, PA) for 2 h at room temperature. After rinsing, sections were mounted and coverslipped with ProLong anti-fade mounting medium (Molecular Probes, Eugene, OR). For labeling, omission of the primary antibody served as a negative control. Labeling for NeuN was viewed under an Olympus BX51 fluorescence microscope (Olympus, New York).

### Quantitative analysis of hippocampal cell loss

Quantitative analysis of hippocampal cell loss was performed in a blinded manned on cresyl violet-stained sections according to previously established protocols [17], [19], [20]. The number of Nissl-stained neuronal profile in areas CA3, CA1, the dentate hilus, and the dentate gyrus were counted in both the right and left hippocampus in horizontal sections for each animal by using four to five Nissl-stained sections that were ∼240 µm apart at the level of the ventral hippocampus. Counting was initiated within the ventral hippocampus at the first point where hippocampal subfields could be easily identified. This level corresponded to horizontal section 54, based on the atlas of Sidman et al. [69]. Hippocampal subfields were based on Franklin and Paxinos classification [64], and numbers within each subfield on both sides were averaged into single values for each animal. Specifically, for dentate hilar cell counts, the hilus was operationally defined as the region bordered by the supra- and infrapyramidal granule cell layers and excluding the densely packed pyramidal neurons of area CA3.

Surviving cells were only counted if they possessed a visible nucleus and characteristic neuronal morphology and had a cell body >10 µm. Counts were performed within the pyramidal cell layer of the CA1 and CA3 areas, in the granule cell layer of the dentate gyrus, and in the dentate hilus. Final neuronal profile counts were expressed as the percentage of cells as compared with intact mice. Six square counting frames (200×200 µm) were randomly placed in the pyramidal layer of fields CA1 and CA3 or in the dentate hilus in four to five regularly-spaced horizontal sections from each animal that were at least 240 µm apart. Only those neuronal nuclei in the focal plane were counted with a 40X objective and considered as a counting unit. Neuronal counting was performed with the aid of Image Pro-Plus software (Media Cybernetics, Inc., Silver Spring, MD) in combination with a SPOT digital camera (Diagnostic Instruments, Inc., Sterling Heights, MI) and a motorized Z-stage (Optiscan, Prior Scientific, Fairfax, VA). All data were expressed as average number of neurons per field and final cell counts are expressed as the percentage of cells as compared with intact mice. Results were assessed statistically by one-way analysis of variance (ANOVA) by using the computer program, SigmaStat (Jandel Scientific, San Rafael, CA) and inter-group differences were analyzed by Student Newman-Keuls post-hoc test.
